# Use of array CGH for molecular characterization of genetic disorders

**DOI:** 10.1186/1755-8166-7-S1-P1

**Published:** 2014-01-21

**Authors:** Ashish Bahal, P Rajitha, Ashwin Dalal

**Affiliations:** 1 Center for DNA Fingerprinting and Diagnostics, Nampally, Hyderabad – 500001, India

## Background

Microarray-based comparative genomic hybridization (array CGH) is a revolutionary platform that has been developed to screen entire genome for copy number variations (CNV) with resolution beyond the capacity of light microscope. ACMG has recommended that array CGH can be used as the first line investigation modality in cases of non-syndromic mental retardation. We present here three cases in which use of array CGH has provided an insight of genetic abnormality.

## Cases studies

Case 1 was a 2 month old child who presented with multiple dysmorphic features of face and extremities. The karyotype of the child showed presence of additional chromosomal material of unknown origin on chromosome 18. Array CGH was carried out which showed duplication of region 2q31.1-q37.3. Whole chromosome paint probe for chromosome 2 showed presence of chromosome 2 material on 18q.

Case 2 was a 10 year old girl having intellectual disability and facial dysmorphism. The initial cytogenetic evaluation did not reveal any abnormality. Further, array CGH showed presence of gain in chromosome 1 from bases 1959715 to 2787707. The segment was analysed using computational software (Decipher) which showed it to be a pathogenic CNV for Intellectual disability.

Case 3 was from two affected children in a consanguineous family with hypotonia and delayed motor milestones. Molecular analysis for Spinal Muscular Atrophy (SMA) was negative. Array CGH was done to look for regions of loss of heterozygosity which were detected in multiple regions. Further analysis of these regions was done to look for the genes associated with SMA and we found PLEKHG5 gene within homozygous regions.

## Conclusions

Our study demonstrates the usefulness of array-CGH in detailed characterization of chromosomal rearrangements, detection of submicroscopic copy number variations and detection of regions of homozygosity in single gene disorders in consanguineous population.

